# English Medicine in the Victorian Era

**Published:** 1897-01-16

**Authors:** 


### The Hospital

A Journal of

The Medical Sciences and Hospital Administration.

Vol. XXI.—No. 538.] [Jan. 16, 1897.

### English Medicine in the Victorian Era.

The present year will witness an event which necessarily has had no parallel in English history—the celebration of the longest, and may we not add, the most illustrious, reign in our annals? It is not, perhaps, competent for any age to pass judgment on itself. Nevertheless, those of us who were not born until some years after the Queen had commenced her reign may, perhaps, consider that we can judge at least a large portion of that reign with a reasonable amount of philosophical impartiality. With regard to medicine in the Victorian era, the one fact which strikes us above all others is that it has gradually, but steadily, worked itself from the lowest level in science and public esteem to a position in the very front rank of the learning and practical progress of the times.

Inevitably the Queen and her reign will be compared with other British monarchs and their eras, and with foreign countries, and those who are now reigning over them. In like manner, in taking a survey of the medicine of the Victorian era, our age will be compared with past ages in our own country, and with the medicine of the present moment and century in all other civilised countries. Can such comparisons be made with understanding, and can they be made with profit? We think they can. English medicine at the present period has a distinguishing note of its own, a note which differentiates it quite decisively from the medicine of our British forefathers; which differentiates it also almost as definitely from the medicine of Germany, France, and other European countries, and, though in a less degree, from the medicine of modern America.

In Great Britain we have our own way of looking at medicine, exactly as we have our own way of looking at domestic politics, colonisation, and the like. In all these things we are nothing if not practical. It is not that we are not physiological, not pathological, not scientific. We are all three, but in an altogether practical sense. Our way is to rule, not to be ruled; and we rule even science as far as we possibly can. If science refuses to be our servant, and to help us in the practical work of life, we are very apt to take her by the shoulders and turn her out of doors without more ado. We do not say that this is what we ought to do; we merely affirm that it is very commonly what in actual practice we do, and cannot help doing. It is a striking and an undeniable fact, and a fact exceedingly well worthy of consideration, that the two circumstances in the medical progress of the Queen's reign which overshadow all others in real and enduring importance owe their origin and usefulness to British sources. When these two circumstances are named, all the world will agree as to their scientific and practical importance. They are the introduction of anæsthetics into medical and surgical practice, and the rise and development of antiseptic medicine and surgery. When we think of what has been done in France, what in Germany, what in America, although we are willing to admit the admirable scientific patience of the Germans, the bright intelligence of the French, and the bold brilliancy of the Americans, we yet feel that in breadth, in solidity, in massive and permanent importance, there are no circumstances in the medical histories of those countries which can with propriety be compared with the bringing of anæsthetics into general use, and the perfecting of the science and art of antiseptic surgery and medicine.

The English medicine of the Victorian era will well deserve the notice of the philosophic historian of the future. In effect it is a new medicine. The old-fashioned British practitioner studied the medical "art," and it was his aim always to do his work *secundum artem*. If the patient did not profit; if more, he died, the practitioner was still satisfied, in that he had done his work *secundum artem*. The modern practitioner, far from subordinating his judgment to the mere rules of an art, carries knowledge, true science, to the bedside of each individual patient, and varies his art according to the detailed condition of that patient. He tests all his work, not by its compliance, or otherwise, with the orthodox conditions of his art, but by the highest and only true standard of "results." The question which the future philosophic historian will ask concerning the new medicine of the nineteenth century is, was it a true medicine; was it based on a true science; was the art the rational outcome of the science; was it intelligently and conscientiously practised? If he is able to answer all these questions in the affirmative he will hardly need to ask the further question, was it productive of the best possible results? Because no other but the best possible results could follow the practice of such a medicine. The philosophic historian, we believe, will be able to answer all these questions in the affirmative, and will be able also to pronounce this general judgment—that the medicine of the Victorian era was as true as it was new, and laid the foundations on which alone all future medicine can honestly and truly be built.

